# Imiquimod as Local Immunotherapy in the Management of Premalignant Cutaneous Conditions and Skin Cancer

**DOI:** 10.3390/ijms241310835

**Published:** 2023-06-29

**Authors:** Emilio Garcia-Mouronte, Emilio Berna-Rico, Belen de Nicolas-Ruanes, Carlos Azcarraga-Llobet, Luis Alonso-Martinez de Salinas, Sonia Bea-Ardebol

**Affiliations:** Dermatology Department, Hospital Universitario Ramon y Cajal, 28034 Madrid, Spain

**Keywords:** imiquimod, immunomodulation, immunotherapy, topical administration, skin neoplasms, actinic keratosis, basal cell carcinoma, melanoma

## Abstract

Cutaneous cancers are, by far, the most common malignant neoplasms of the human being. Due to the great array of clinical conditions, their worldwide increasing incidence and the steady ageing of the population, non-invasive treatments modalities that show a good clinical response, a proper benefit–risk ratio and cosmetic results are becoming increasingly important in the clinical setting. Imiquimod is a topically applied immunomodulator which is often used in the management of several premalignant and malignant cutaneous disorders. This article is a review of the current literature on its mechanism of action, pharmacokinetics, and therapeutical effects.

## 1. Introduction

Cutaneous cancers are by far the most common neoplasms in humans, comprising a third of all diagnosed malignancies [1,2,3,4]. Approximately 10–30% of individuals will develop skin cancer during their lifetime, which poses a serious challenge for healthcare systems [5,6]. For instance, the annual cost of treating skin cancer in the United States is estimated at USD 8.1 billion and is growing faster than that for any other type of tumour [6,7].

Basal cell (BCC) and squamous cell carcinomas (SCC) comprise up to 80% and 20% of nonmelanoma skin cancers (NMSC) or keratinocyte cancers, respectively [2,5]. At least 80% of NMSC cases appear in patients older than 60 years of age [2,8]. The number of new diagnosed cases of NMSC has increased by an annual rate of 3–8% during the last decades. For instance, in the United States alone, their incidence has more than tripled between 1984 and 2014 [6,9]. Different causes have been proposed to explain this phenomenon, such as iatrogenic immune suppression and an increased exposure to ultraviolet (UV) radiation due to a longer lifespan, more frequent outdoor activities, and ozone layer depletion [4,8,10].

Due to their easily detectable nature, prompt diagnosis and local management are usually achieved [5]. Despite their high overall survival rate, NMSC are associated with considerable morbidity due to disfigurement and functional impairment [5].

The management of NMSC comprises a wide array of therapeutic options (Table 1) [7,11]. Complete eradication of the tumour is the final and most important outcome [12]. Surgical removal, preferably via the simplest method, is still the gold-standard treatment for most skin cancers, with a cure rate greater than 90% [2,3,4,5,13]. Nonetheless, the preservation of function and cosmesis should also be taken into consideration in multiple-location (i.e., genodermatoses or immunosuppressed individuals) or critically located tumours (i.e., face) [5,14].

The choice of treatment is thus based on the expected outcome, objective tumoral parameters (histological subtype, size and anatomical location), cost of treatment, the patients’ preferences, general health conditions, and estimated life expectancy [8].

Topical therapies are reserved as intentional healing therapies for low-risk tumours (i.e., small and superficial), although they can also be employed as palliative strategies in patients with a high morbidity index or in cases where surgical resection is not feasible or is contraindicated [11,16].

Among topical therapies, immune response modifiers (IRM) stand out for their direct and indirect stimulation of antitumor innate and adaptative immune responses, tissue-sparing and function-preserving properties [1,5,14,19]. Imiquimod (IM) is the most used topically applied IRM and was first approved by the Food and Drug Administration (FDA) in 1997 for the treatment of adult external genital and perianal warts [1,4,20]. Indications for head and scalp non-hypertrophic actinic keratoses (AK) and non-head and neck superficial basal cell carcinoma (sBCC) were added in 2004 [1,11,13,21]. Since then, it has been employed off-label for different infectious and neoplastic superficial skin disorders, such as Bowen’s disease (BD), nodular basal cell carcinoma (nBCC), SCC, lentigo maligna (LM), melanoma metastases, cutaneous T-cell lymphomas and pyogenic granuloma [1,2,21,22,23]. However, the scientific evidence supporting its use in these latter conditions is anecdotical and relies mostly on case series and open-label trials, with varying and inconsistent treatment regimens [24].

Despite its frequent use by dermatologists, the physiologic pathways involved in the therapeutic action of IM remained elusive in the first years after its approval. This “enigma” has been partially resolved due to the publication of several articles reporting the effects of IM on skin cancer cells [10,23,25,26,27]. For these reasons, the aim of this review is to better define the molecular mechanisms of action of IM and its indications in cutaneous neoplastic disorders.

## 2. Chemical Structure and Pharmacokinetics

The chemical structure of IM is 1-(2-methylpropyl)-1H-imadazo[4,5-c]quinolin-4-amine (imidazoquinoline) [1,14,28]. This small molecule (240.3 Da) and nucleoside analogue was initially discovered in a programme to develop inhibitors of herpes simplex virus replication [6,10,14,28,29,30].

IM is commercially available as an oil-in-water-based 3.75–5% varnishing cream in sachets [11,31]. Manufacturers recommend its application at bedtime [28]. No more than one sachet should be applied to a contiguous area during each application [11,31]. While treating periocular tumours, it is suggested to apply the product with a swab onto the lesion to avoid contact with the cornea or conjunctiva [20]. Occlusion should be avoided since it does not increase efficacy and causes more severe local reactions [1,14,32]. Despite IM lacking the potential for inducing phototoxic and photoallergic reactions, the exposure to UV radiation should be minimized because of an increased sunburn susceptibility secondary to the vehicle [11,14]. Consequently, the site of treatment should be cleaned with soap and water 8 h afterwards [11,31]. Patients need to wash their hands before and after its use [11,31].

Therapeutic regimens are individualized according to clinical and/or histological diagnosis, the severity of the condition and expected tolerance and compliance by the patient [14]. The frequency of use is highly variable and may be daily with rest periods, 2–3 times/week [14]. etc. The duration of the treatment commonly ranges from 6 to 16 weeks [1].

Despite minimal systemic absorption, with a median bioavailability from 1% (one-two sachets, five times/week) to 3% (six sachets, five times/week), IM is still classified as a pregnancy category C drug [14]. Thus, contraception is encouraged for women of childbearing age while on treatment [11]. In relation to other special populations, it is unknown whether IM is excreted in the milk [1]. In contrast, its safety in paediatric subjects aged 2–12 years has been assessed in double-blind RCTs [14].

## 3. Mechanisms of Action

UV-induced skin carcinogenesis mostly relies on two mechanisms:

- DNA damage. Chronic UV exposure leads to the accumulation of DNA mutations that surpass the physiological repair mechanisms [4,22]. Whereas UVA (320–400 nm) causes indirect genetic damage through photooxidative stress, UVB (290–320 nm) directly induces the formation of thymidine dimers and C-T/CC-TT conversions [33].
- Impaired T-cell immune surveillance, either locally through the reduction in and inactivation of Langerhans cells (LC), or systemically by skewing the differentiation of T helper cells to an immunosuppressive phenotype [29,34].

Immunosurveillance is vital for the survival of malignant cells [28]. Tumours develop different mechanisms to escape recognition by immune cells, such as the following:

- Reduced expression of major histocompatibility complex (MHC) I, preventing antigen presentation [4].
- Generation of an immunosuppressive tumoral microenvironment through the liberation of pro-tumoral cytokines (i.e., IL-10 and TFG-β) and the recruitment of CD4^+^CD25^+^FoxP3^+^ regulatory T cells, myeloid-derived suppressor cells, N2-polarized neutrophils, tumour-associated macrophages and tolerogenic dendritic cells (DC) [4,34,35].
- Resistance to apoptosis [4].

For these reasons, therapeutic agents, such as IM, that simultaneously bypass tumoral resistance to apoptosis and stimulate immune recognition have a considerable clinical benefit in the management of cutaneous malignancies [28].

Depending on the molecular target, the effects of IM can be divided into TLR7-dependent and TLR7-independent.

### 3.1. TLR7-Dependent Effects

TLR7 plays an important role in recognizing pathogen-associated molecular patterns (PAMPs) [14,35]. This membrane receptor is mostly found in macrophages, monocytes, DCs and LCs, although it can be also expressed by other immune cell types [5].

IM mainly binds TLR7, although it can also serve as a TLR8 analogue in high concentrations [28,35]. TLR7-IM binding triggers a MyD88-dependent signalling cascade, recruiting protein kinases and ultimately stimulating the NF-κβ transcription factor, enhancing the transcription of numerous pro-inflammatory genes [1,14,19,28].

The effects of IM are thus pleiotropic, strongly activating the innate immune system while providing a link to the adaptative immunity [1,14]:

- The innate immune system is the first line of defence against non-specific infectious pathogens and different physical or chemical insults [1]. Several cell types (neutrophils, eosinophils, natural killer (NK) cells, basophils and mast cells) participate through phagocytosis, chemokine synthesis and inflammatory mediators [1].
- Epidermal and dermal plasmacytoid dendritic cells (pDC) are the primary skin cell population responsive to IM since they are stimulated in vitro using lower doses than other cell types [14]. IM specifically induces their functional maturation and migration to regional lymph nodes, which is essential for triggering a profound tumour-directed T cell response [14,28,34].
- After pDC, macrophages are one of the cell lines more sensitive to this IRM [14]. IM not only stimulates the survival of macrophages through the upregulation of potent apoptosis inhibitors, such as Fas-associated death domain-like IL-1β-converting enzyme inhibitory protein (FLICE), but also strongly activates their function through the upregulation of macrophage inflammatory proteins (MIP)-1α, MIP-1β, IL-1α, nitric oxide synthase (NOS) and CD40 [14].
- IM has been demonstrated to stimulate the synthesis of IFN-α, IFN-γ, TNF-α, IL-1a, IL-2, IL-6, IL-8, IL-10, IL-12, G-CSF and GM-CSF via macrophages and DC [1,5,14,19,28]. These molecules (specially IFN-γ, IL-12 and TNF-α), together with LC, skew naïf T cell differentiation towards a Th_1_ phenotype, fostering a potent and antigen-specific adaptative immune response against tumour-associated antigens (TAA) [1,5,14,19,28].
- Interferons play an essential role in the antitumoral effects of IM [4,19]. IFN-α2a and IFN-α2b inhibit the growth of malignant cells and increase the expression of IL-12βR in CD4+ T cells [4,19]. The activation of this receptor leads to an additional synthesis of IFN-γ by naïve T cells [19]. Berman et al. [26] showed that after IFN-α treatment, BCC cells expressed FasR. FasR-FasL binding can occur after BCC cell– and/or BCC cell–T-cell interaction and activates the apoptotic extrinsic pathway [26]. Even a suicidal activation of FasR by BCC cells co-expressing FasR and FasL may happen [26].
- IM also upregulates vital cytokines (i.e., CCL5, CXCL9, CXCL10) for homing T cells [25]. After 3–6 days of treatment application, a brisk lichenoid and peritumoral inflammatory infiltrate consisting mainly of CD45RO+ T lymphocytes, DC and macrophages develops [2,14,24,29]. Afterwards, the peritumoral and intratumoral macrophage count increases [14].
- IM also enhances the antigen’s further presentation process to T cells through the upregulation of costimulatory membrane receptors in antigen-presenting cells (APC), such as CD40, CD80, CD86 and ICAM1, and the expression of MHC I and MHC II [1,14]. Increased expression of MHC-I has also been confirmed in BCC cells [4].
- NK cells can also respond to IM [14,28,34]. For instance, it induces the expression of 2′5′-oligoadenylate synthetase and NOS [14,28,34].
- Additional mechanisms through which IM can hamper tumour growth and dissemination have been described [14,34,36]. It has shown clear antiangiogenic mechanisms by increasing the synthesis of anti-angiogenic molecules (IL-10, IL-12, tissue inhibitor of matrix metalloproteinase (TIMP), thrombospondin 1 and 2 (TSP-1/TSP-2)) and simultaneously downregulating the expression of pro-angiogenic factors (basic fibroblast growth factor (bFGF), matrix metalloproteinase-9 (MMP-9), vascular endothelial growth factor (VEGF), angiogenin and IL-8) [14,34,36]. This could be useful in neoplasms with a considerable formation of vessels, such as pyogenic granuloma, Kaposi’s sarcoma, infantile haemangioma and angiosarcoma [34].
- After exposure to IM, the levels of MMP inhibitors (TIMP-1 and TIMP-2) are increased 14- and 5-fold, respectively, [34]. The cleavage of collagen IV by MMP is essential for local malignant invasion and systemic dissemination [34].
- Interestingly, IM inhibits IL-13 signalling, which is over-stimulated in most malignant neoplasms [5,25,28].

These immune effects correlate with the clinical findings observed in the RCTs and case series [32,37,38,39,40,41]. Whereas the initial intense inflammatory response within the first days of treatment depends on the activation of the innate immune system, the continuing improvement after treatment discontinuation (i.e., AK) might be secondary to the reversal of local immunosuppression of chronically sun-damaged areas, thus leading to a persistent and protective antitumoral skin Th_1_-skewed immunity (“vaccination effect”) [1,14,28].

### 3.2. TLR7-Independent Effects

It was initially though that the mechanism of action of IM relied only on the stimulation of the immune system [1]. This assertion was called into question when various authors reported the clearance of cutaneous lesions after treatment without clinically evident inflammatory signs [14]. Biopsies taken from BCC and AK after the discontinuation of IM confirmed the preservation of non-neoplastic cells [26]. Had its mechanism of action been entirely dependent on immunomodulation, the surrounding normal cells would have been damaged by the inflammatory infiltrate [29].

Since then, several works have been published that confirm that IRM displays direct antineoplastic activity:

- Impaired viability of neoplastic cells [29]. Schön et al. [29] detected a mean reduction in cell count of 40–70% after SCC and HaCaT lines were cultured with IM 50 µg/mL. The proapoptotic effect was dose-dependent [29].
- Disruption of the electron transport chain through the inhibition of the mitochondrial complex and cytosolic NQO2, facilitating electron leakage and robust production and accumulation of ROS [23]. The mitochondrial membrane collapse leads to ATP depletion, mitophagy and, ultimately, cell death [23].
- Mitochondrial fragmentation through dynamin-related GTPases, such as MFN1/2, OPA1 and DRP1, facilitating mitophagy [23].
- Activation of inflammasome, leading to increased synthesis of IL-1β and IL-18 [14,23].
- Inhibition of adenosine intracellular receptors in clinical dosing settings, showing the highest affinity for A_1_ and A_2A_ subtypes [28]. This blocks an immunosuppressive feedback which strongly activates proinflammatory pathways [14,28].

These phenomena are more dominant in skin cancer cells than in normal keratinocytes [23]. Among these effects, the induction of autophagy is considered one of the most relevant mechanisms of action of IM [35]. Autophagy is a cellular response to bioenergetic stress that permits cell survival via a dual mechanism [10,27,35]:

- Engulfment of large cytoplasmic portions containing damaged organelles and long-lived macromolecules within double-membrane autophagosomes, subsequently fusing with lysosomes [10,27,35]. This leads to considerable internal remodelling and helps in maintaining the proper quality of the mitochondrial population [35].
- Generation of glycolytic substrates for ATP synthesis [10].

Autophagy is regulated by a family of highly preserved genes known as the ATG family and can be activated via the following processes [23,27]:

- ER-stress/PERK/PKR axis through ROS-dependent manner [23].
- Release of cathepsins B (CTB) and D (CTD) into the cytosol [27]. Massive ROS production induces lysosomal membrane peroxidation, affecting its integrity and increasing its permeability [27]. The release of cathepsins lowers the cytosolic pH and activates additional hydrolases, leading to the indiscriminate digestion of cellular components and, ultimately, to autophagic apoptosis [23,27]. If severe, it could result in uncontrolled cell necrosis [27].

Autophagy plays a dual role in cancer cells depending on the cell type and therapeutic mechanism of the drug [23]. For instance, IM-induced autophagy in APC accelerates the elimination of intracellular antigens and fosters the innate immune response [29,35].

**Figure 1:**
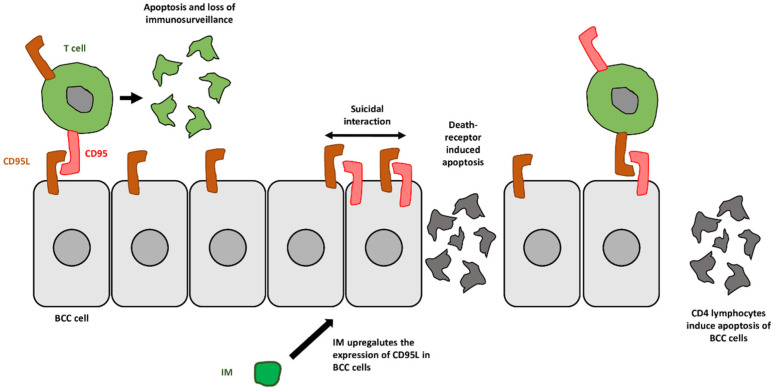
Extrinsic pathway of apoptosis induced by imiquimod (IM). Under normal conditions, basal cell carcinoma cells (BCC) lack CD95, which allows them to elude immunosurveillance. IM upregulates the expression of CD95 and CD95L in BCC cells, triggering the extrinsic pathway of apoptosis through BCC cell–BCC cell and BCC cell–CD4^+^ T cell contact.

Apart from these, IM shows noteworthy proapoptotic effects in clinical dosing settings, even in the absence of immune cells, overcoming the resistance of neoplastic cells to death signals [28]:

Extrinsic pathway (death-receptor induced apoptosis) [27]:

- The longevity of BCC cells is due, at least in part, to the absence of CD95 [26]. On the other hand, these cells strongly and diffusely express CD95 ligand (FasL), which is involved in the apoptosis of infiltrating antitumoral T cells, allowing the BCC to escape the host’s immune surveillance [26].
- IM stimulates the expression of membrane-bound death receptors in sBCC cells, such as CD95 and CD95L (FasR, Fas-APO1 receptor system) [14,28,29]. CD4+ cells can trigger the apoptosis of malignant cells through CD95-CD95 ligand binding [4]. When this occurs, a signalling cascade ensues, which ultimately results in DNA fragmentation, cell–membrane blebbing and the expression of phagocytosis signalling molecules on the cell surface (Figure 1) [26]. These effects have been confirmed in vivo by Berman et al. [26], who excised 10 non-head primary BCC immediately after treatment with either IM 5% or placebo, applied five times/week for two weeks. The histological clearance rate was 80% in IM-treated BCC (vs. 0% in the placebo group) [26]. The expression of CD95 in BCC cells was 75% in IM-treated patients (vs. 0% in the placebo group) [26].
- Nevertheless, the expression and activation of CD95 and TRAIL receptors R1-R4 in SCC cell lines do not significantly change after exposure to IM [29].

**Figure 2:**
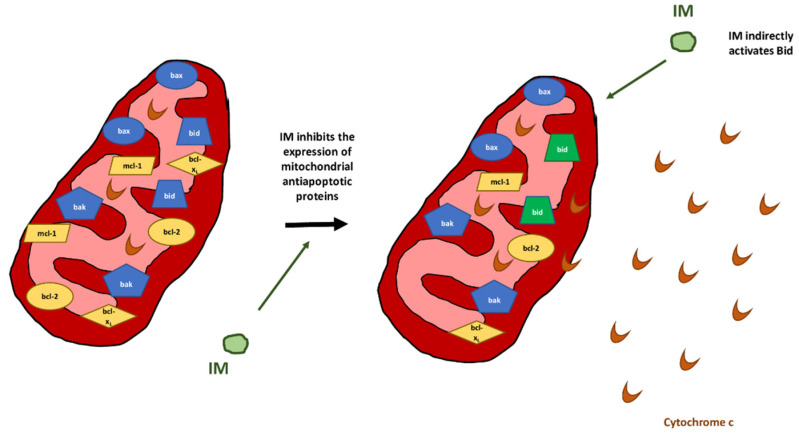
Intrinsic pathway of apoptosis induced by imiquimod (IM). IM induces a disbalance between mitochondrial proapoptotic (blue: bax, bak, bid) and antiapoptotic (yellow: bcl-2, bcl-x_L_, mcl-1), favouring the translocation of cytochrome c into the cytosol. This leads to the activation of caspases and, ultimately, cell death.

Intrinsic pathway (chemically induced apoptosis) [29]:

- It is the main apoptotic mechanism in SCC and melanoma cells, although it has been observed in BCC as well [10,27]. As a death-receptor-independent apoptosis pathway, its role in IM-induced apoptosis is critical since its inhibition in vitro with Z-IETD-FMK leads to increased cell viability [27].
- This pathway is mainly triggered by the bcl-2-dependent release of mitochondrial cytochrome C into the cytosol [14]. Then, cytochrome C binds APAF-1 and pro-caspase-9, building apoptosomes, which further activate caspase-9 and caspase-3 [28,29]. This has been confirmed in BCC cell lines [28,29]. Caspases are essential in IM-induced apoptosis, since the in vitro use of pan-caspase inhibitors completely abrogates it [28,29].
- The translocation of cytochrome c depends on the ratio between antiapoptotic (bcl-2, mcl-1, bcl-x_L_) and proapoptotic (bax, bak, bid) mitochondrial membrane-bound proteins [28,29]. IM dramatically and rapidly inhibits the translation of bcl-2, mcl-1, bcl-x_L_ and other antiapoptotic proteins in BCC cells (Figure 2) [10,14,28]. It has been shown that IM blocks the initiation and elongation phases of mcl-1 translation by decreasing the levels of phosphorylated 4E-BP1 and stimulating the phosphorylation of eEf2 [10].
- CTSB and CTSD, whose release into the cytosol is induced by IM, activate the proapoptotic protein Bid [27]. This increases the permeability of the mitochondrial outer membrane, causing cytosolic translocation of cytochrome c, inhibition of mitochondrial complex I and a decrease in mitochondrial membrane potential [27].
- CTSD indirectly activates effector caspases (caspase-3 and caspase-7), which, in turn, target proteins involved in the apoptotic response [27,29]. Importantly, the activation of caspase-3 has been confirmed in SCC cell lines after treatment with IM, increasing the pro-caspase-3/caspase-3 ratio to 10:1 compared with that of vehicle-treated cultures [27,29].
- Downregulation of antiapoptotic genes (hurpin and HAX-1) in AK cells [28].
- Oncogenic signalling modulation:
- Downregulation of several MAPK-related genes (MAP2K4, MAPK1, MAPK11 and MAP3K5) in BSM [25].
- Inhibition of Hedgehog signalling through adenosine receptor/protein kinase A-mediated GLI phosphorylation [34].

In conclusion, IM fosters a potent tumour-directed response through the activation of simultaneous and synergistic antineoplastic pathways [14].

## 4. Clinical Indications

### 4.1. Actinic Keratoses

AK are intraepithelial dysplasias that involve the basal cell layer and can additionally extend to the overlying strata [33]. They consist of proliferating atypical keratinocytes with large nucleus/cytoplasm ratios, hyperchromatic nuclei, marked nuclear and cell pleomorphism, disordered terminal differentiation and a loss of polarity [33].

They are the most frequent carcinoma diagnosed in situ in humans [24,33]. AK arise in chronically light-exposed areas (face, back of the hands, and scalp in bald individuals) and are thus associated with cumulative lifetime sun exposure (i.e., outdoor work) [8,14,33,42]. Their incidence has continue to increased over the past few decades [33]. The prevalence of AK in the United Kingdom is estimated to be 34% in males and 18% in females older than 70 years of age [33]. Immunosuppressed hosts have a 250-fold risk of developing AK, with at least 40% of affected individuals progressing to invasive squamous cell carcinoma [33]. Given its high prevalence, AK pose a considerable burden for healthcare systems [43]. Their diagnosis and treatment cost in the United States surpasses USD 1 billion dollars annually [43].

Clinically, AK are defined as multiple red-to-brown dry and rough macules or papules, ranging from a few millimetres up to 2 cm [33]. Sometimes, they are covered by an overlying hyperkeratotic scale [33].

AK are the best clinical indicator for the development of future cutaneous malignant neoplasms, especially SCC [42]. They are indeed regarded as the initial stage of a biological continuum that ranges from AK to BD and SCC [5]. These entities share mutations (p53, expression of telomerases) and chromosomic aberrations [33].

Nevertheless, the clinical behaviour of AK is unpredictable [43]. Whereas some lesions tend towards spontaneous regression (approximately 25% per annuum) or persistence without further changes, others convert to truly invasive carcinomas [43]. The annual progression rate to SCC has been recently estimated to range from 0% to 0.5% per lesion-year [43]. The risk seems to be lower in individuals with no history of previous NMSC [44]. Overall, the clinical factors for predicting the subsequent progression of AK are unfortunately not well-defined [5,44]. Given this uncertainty, every patient suffering from AK should be offered prompt and appropriate management [33,44]. AK are often managed in the clinical setting as chronic disorders, requiring different and even the repetition of distinct treatment modalities over the course of time [44,45].

IM is approved as a field cancerization treatment for the management of scalp and facial non-hypertrophic AK located in a contiguous area measuring 25 cm^2^ or less [1,31]. Formulations of 2.5%, 3.75% and 5% are approved by the FDA [6]. Less concentrated formulations display similar efficacy with higher tolerability [6].

It must be applied once to three times per week for one to four months [11,14,18,31]. Additional doses could be prescribed to patients with an incomplete clinical response [11]. The duration and frequency are usually individualized according to the number of lesions and the severity of the disease, although treatment should not be extended for missed doses [1,14].

Short-term complete clearance at 3–6 months varies between 17.4–39%, with a mean reduction in lesion counts ranging from 55 to 86.6% [11,37,40,42,46,47,48,49,50,51]. At least 59% of patients experience a reduction in AK lesions by more than 75% [1]. The preventive potential effect against new clinically evident AK declines after the discontinuation of the treatment, practically disappearing 9 months afterwards, with a relapse rate of 17.4–39% and a lesion count near 50% of the baseline numbers [42,45].

Similarly to 5-fluorouracil (5-FU), IM may unmask subclinical preneoplastic changes during the first weeks of treatment in up to a half of patients, which is not associated with a worse final clinical outcome [1,22,45].

Nonetheless, IM is not the most efficacious treatment for preventing the progression of AK into SCC [44]. In a single-blind multicentre RCT in the Netherlands, the risk of developing SCC in the following four years after different field cancerization-directed treatments was assessed [44]. Immunocompetent patients older than 18 years of age with Fitzpatrick’s phototypes I-IV and at least five AK lesions at the initial visit within a treatment area of 25–100 cm^2^ were included [44]. A total of 156 patients were treated with IM 5%, three times/week, for 4 weeks [44]. The risk of developing SCC was 5.8% in patients treated with IM, which was higher than that in patients initially treated with 5-FU [44].

On the other hand, since there is a wide array of field cancerization-directed treatments with distinct and specific mechanisms of action, the combination of IM with other therapeutic options (photodynamic therapy (PDT), 5-FU, tirbanibulin, diclofenac) may display synergistic effects that could ultimately lead to improved clinical and histological outcomes [39,52,53]:

- IM + PDT. According to the available literature data, there are only two prospective trials where these two strategies were simultaneously or sequentially employed in the management of AK [52,53].
  o Sequential regimen. Pre-treatment with PDT may have several advantages: it reduces the lesion count, possibly increasing the tolerability of IM; and generates a residual inflammatory response that may bolster IM-induced stimulation of the immune system, thus increasing its efficacy [53]. Shaffelburg [53] performed a double-blind, vehicle-controlled, split-face clinical trial of 25 patients with at least 10 facial AK. They were first treated with PDT (20% 5-ALA, blue light, two monthly sessions), followed, one month later, by the application of IM 5% cream (two times/week, 16 weeks) only to a single half of the face [53]. The median AK lesion reduction at month 12 was higher in the sequential treatment group (86.7% vs. 73.1%, *p* = 0.0023) [53]. The adverse reactions reported were not severe [53].
  o Simultaneous regimen. Tanaka et al. [52] conducted a single-centre clinical trial where 18 patients with AK on the face, head and scalp where randomly allocated to receive 5% IM cream (three times/week for one month), PDT (20% 5-ALA PDT, red light, 50 J/cm^2^, once/week for three weeks) or a simultaneous combination of both treatments (5-ALA PDT, 50 J/cm^2^, every Monday for three weeks + IM 5% cream, every Wednesday and Friday for one month) [52]. The patients were clinically assessed one month after treatment discontinuation [52]. The clinical clearance rate was higher with IM+PDT (100% vs. 66.7% (IM) vs. 47.1% (PDT), *p* < 0.05) [52]. Adverse events were mild to moderate, and no statistically significant differences were found between the three groups, either in their incidence or in their severity [52].
- IM + 5-FU.
- To the best of our knowledge, the benefits of this specific combination in the management of AK have only been assessed in one single-centre open-label study [39]. A total of 64 patients with extensive AK on the face, scalp, upper limbs, or legs were concomitantly treated with three courses of 5-FU 5% cream (once daily in the morning, for seven days) and IM 5% cream (once daily in the night, for six days), with a hiatus of three to four weeks between each cycle [39]. A total of 25% of participants withdrew from the study, although the authors reported that only two patients (3.13%) abandoned the study because of side effects [39]. Interestingly, adverse reactions were rarer and milder in the second and third cycles, which could be secondary to a reduction in lesion count [39]. Treatment breaks were deemed essential to improve the tolerability of the combination and secure a proper compliance [39]. The authors claimed this technique was beneficial since the total duration of the regimen was still shorter than those commonly used in monotherapies [39]. Nevertheless, the lack of clinical variables (objective lesion count), comparison groups and histological assessment clearly affects the validity of their observations [39].
- Interestingly, Nahm et al. [54] recently published the results of a single-centre retrospective review of 327 patients with AK on the face or ears who employed a combination of IM 5% cream, 5-FU 2% solution and tretinoin 0.1% cream [54]. The participants applied the mix up to 30 times within a 76-day period at their discretion, at a maximum frequency of five times/week for six weeks [54]. They were instructed to individualize the frequency for mitigating excessive irritation [54]. One year after the discontinuation of the treatment, the risk of in-field (OR = 0.06, 95% CI [0.02, 0.15]) and out-field NMSC (OR = 0.25, 95% CI [0.14, 0.42]) was dramatically inferior to that in the year before field-treatment [54]. The participants required fewer sessions of cryotherapy for managing AK (2.3 vs. 1.5, *p* < 0.001) [54]. Notwithstanding these excellent results, there were several biases: a retrospective nature, limited post-treatment follow-up and an undetermined AK count [54]. Future prospective studies and RCT are warranted to confirm and better understand these findings.
- There are no RCT that evaluate the efficacy of combination treatments of IM with tirbanibulin or diclofenac.

Moreover, the effectiveness and tolerability of the combination of IM with lesion-directed treatments has also been studied, especially with cryotherapy [55]. In a multicentre vehicle-controlled double-blind RCT, 247 patients with at least 10 typical facial AK were randomly treated with IM 3.75% cream (once daily for two weeks on the treatment, two weeks off the treatment, and once daily for two weeks on the treatment (two–two–two regimen)) or placebo [55]. At the first visit, a minimum of five AK were treated with cryosurgery in every participant of both groups according to the investigator’s usual clinical practice [55]. There was a greater median percent reduction in lesion count at week 26 for the cryosurgery/IM group (86.5% vs. 50%, *p* < 0.0001) [55]. However, a considerable limitation of this study was the absence of a comparison between cryotherapy+IM and IM in monotherapy [55].

### 4.2. Bowen’s Disease

BD is an intraepithelial dysplasia where the stratum basale is preserved, leading to a “horizon” appearance under a microscope [14]. It clinically manifests as enlarging erythematous, desquamative and often well-defined plaques [14].

The surgical treatment of BD is challenging due to its common location in difficult-to-treat areas (i.e., shins), extension and multifocal nature [14]. Thus, IM could serve as an adequate medical treatment [11]. Nevertheless, the scientific evidence is low and mainly consists of case reports [1,2,5,11,14,31]. Different regimens have been used (once daily, three times and five times per week for 3–20 weeks). The overall clearance rate ranges between 57 and 80% [1,2,5,11,14,31]. Thickness lesion and hyperkeratosis are associated with a poorer response [11].

### 4.3. Basal Cell Carcinoma

BCC is the most common human malignant neoplasm and the tumour with the highest mutational burden [6,14]. In total, 4.3 million cases of BCC are annually diagnosed in the U.S [6]. Its age-standardized incidence rate in Australia was set at 770 per 100,000 person years [6]. BCC incidence increases annually by 2–10%, especially in young women [3,9,34]. Intermittent and high UV exposure during recreational activities (i.e., sunburns during childhood and adolescence) is deemed to be the most important factor in the carcinogenesis of BCC [8]. They are most commonly located in the head and neck (70%) of middle-aged/elderly light-skin individuals [3,6,30]

Although its growth rate is usually slow, its clinical behaviour remains unpredictable [3,56]. If left untreated or inappropriately managed, BCC may cause considerable morbidity through the local invasion and destruction of surrounding tissues [3,6]. Nevertheless, it seldom metastasizes (0.0028–0.55%) [3,6,30].

Several histological subtypes have been described, with the most important being the following: superficial (sBCC, 20%, the commonest subtype in Australia), nodular (nBCC, 50–79%), infiltrative, morpheaform (5–10%), cystic, metatypical and basosquamous [3,6,8,30,57]. The histological classification is a key factor in deciding which is the most appropriate treatment for the patient since the cure and relapse rates differ between variants [6]. Consequently, depending on the clinical and histological patterns, BCC can be divided into two main risk categories (Table 2) [3].

As a non-surgical therapy, IM is reserved for the management of low-risk BCC where the control of histological margins is less important [3]. The application of the product should encompass a margin of 1–3 cm perilesional normal-appearing skin [24].

Most RCTs excluded sBCC and nBCC in immunosuppressed hosts, tumours with a surface area larger than 2 cm^2^, BCC located in certain areas (anogenital region, hands, feet, within 1 cm of the hairline, eyes, nose, mouth or ears) and previously treated cases [1,3,57].

**sBCC:**

- IM is the most efficacious FDA-approved treatment for sBCC and is the preferred modality in low-risk areas [2,6]. sBCC is more responsive to IM than nBCC [1,5,6,14]. Only the 5% formulation is licensed [6].
- Different regimens have been employed: twice daily, once daily, and every other day for 6–16 weeks [15,58]. IM presents a clear dose–frequency relationship in the management of BCC [1,5,32,59]. Histological clearance at week 12 was complete if the patients were treated twice daily [14]. When the frequency was reduced, the rate progressively decreased to 82% (five times/week) and 52% (three times/week) [6,14]. A once-daily dosing, five days/week, for six weeks is the regimen approved by the FDA since it achieves a good complete clearance rate (81–90%) with an adequate safety profile [1,6,14,60].
- Overall clinical and histological clearance rates at 3–12 months range from 60% to 80% in well-defined RCTs [15]. The cure rates with varying treatment regimens, from twice daily to twice weekly, and with follow-ups between 12 weeks and 5 years range from 43% to 94% [12].

**nBCC:**

- IM is used off-label in the management of nBCC [6]. The preferred regimen (five times/week for 12 weeks) shows a clearance rate of 50–65% [2,3,12,32]. Therefore, the overall efficacy of IM in nBCC is poor, with at least a third of the patients presenting residual disease after treatment discontinuation [11,61].
- Given these results, it has been hypothesized that pre-treatment with cryotherapy could enhance tumour immunogenicity and even provide a clinical benefit in BCC refractory to IM in monotherapy [62]. Messeguer et al. [62] selected 23 BCC (sBCC = 11, nBCC = 12), 1 to 2 cm in size, resistant to IM 5% cream in monotherapy, administered five times/week for six weeks [62]. Cryotherapy was applied one month after the completion of the initial therapy [62]. Beginning the same day, the participants applied a second cycle of IM 5% cream with the same dosing regimen [62]. The complete clearance rate at one month was 83%, which was still lower in nBCC (67% vs. 91%) [62]. Only one relapse was detected in the follow-up period (at least one year) [62]. Four tumours still required an additional cycle of cryoimmunotherapy [62]. However, these results have several limitations: the study was open-label and lacked a control group, the follow-up period was limited, and a complete cure was not confirmed via biopsy [62].
- Additionally, IM can be employed as an adjunctive therapy to Mohs’ surgery, electrodessication and curettage [8,14,63]. For instance, in an open-label uncontrolled single-site study, 14 patients with high-risk BCC (>2 cm) unfit for surgery, chemotherapy and radiotherapy were sequentially treated with initial vaporization with aCO_2_ laser in ablative mode (initial parameters: 600 µs pulse duration, 45 mJ energy, repeat time 10 ms, stack level 2), followed by cycle therapies of diclofenac sodium 3% gel (once daily for five days) plus IM 5% cream (once daily for two days), up to a maximum of 24 weeks [64]. Nine patients relapsed during the treatment period [64]. Despite these interesting approaches, surgical excision remains the gold-standard treatment [15]. For instance, Sinx et al. [65] directed a multicentre noninferiority clinical trial where 145 immunocompetent patients with histologically proven primary nBCC of 4 to 20 mm, with or without a superficial component, were randomly assigned to be treated with surgical excision with a 3 mm safety margin, or curettage followed by treatment with 5% IM (five times per week, six weeks) [65]. The patients underwent clinical and dermoscopic assessment one year after the discontinuation of the treatment [65]. If treatment failure was suspected, a punch biopsy was carried out to confirm tumoral relapse [65]. One year after treatment, the relapse rate was superior in the group of curettage+IM (13.7% vs. 0%, *p* = 0.0004) [65]. Nonetheless, it should be highlighted that approximately a quarter of the patients (23.7%) treated with curettage+IM did not fully comply with the regimen [65]. No differences were detected as regards severe pain (13.5% vs. 27%, *p* = 0.208) [65]. However, the investigator-reported cosmetic outcome was superior [65].

### 4.4. Lentigo Maligna

Lentigo maligna is an in situ phase of melanoma which arises in chronically sun-damaged skin areas [14]. Since malignant cells are restricted to the epidermis, its metastasizing potential is limited [6,66]. It accounts for approximately 80% of all melanoma in situ (MIS) [66].

Its incidence is higher in elderly individuals, and it is most frequently located on the face [66]. A total of 53,120 new cases of MIS were reported in 2009 [66].

Although surgical excision is still considered the gold-standard treatment, its commonly large size at diagnosis and the patients’ comorbidities make non-invasive treatment modalities good alternatives for the management of this condition [66]. Since melanoma is one of the most immunogenic malignancies, IM could theoretically serve as an appropriate antineoplastic treatment [67]. IM is in fact reserved as a third-line treatment for cases in which surgical excision or radiotherapy are not feasible, such as in elderly and/or fragile patients [6,14,17,66]. The clearance rates reported with IM have ranged from 66% to 100% [66]. The guidelines do not specify the optimal dosing, schedule or length of treatment [6,11,66]. However, reviews that assessed the outcomes of non-surgical therapies for LM recommended at least 60 applications, six to seven times/week [68,69]. Large controlled RCTs with long follow-up periods are nevertheless needed to define the best dosing regimen [6]. On the other hand, as the effects of IM are non-ablative, a hypothetical risk of local recurrence and progression to invasive melanoma exists [14]. These doubts are mainly raised by the difficulty in objectively assessing histological clearance after treatment [66].

Additionally, IM can be used as an adjuvant treatment to other therapeutic approaches, such as surgery or radiotherapy [6,11,35]. In this sense, Cho et al. [35] studied the synergistic effect of IM in radiotherapy-treated murine melanoma cell lines B16F1 and B16F10. After incubation for 24 h, an increase in autophagy-associated proteins was detected [35].

### 4.5. Melanoma Skin Metastases

The management of metastatic melanoma is extremely complex [1]. Patients commonly require the combination of different treatments to better control the burden of the disease [14]. It has not been demonstrated whether the treatment of melanoma cutaneous and subcutaneous metastases has an impact on overall survival [1].

Although it can eradicate accessible dermal metastases, IM does not treat subcutaneous metastases and does not prevent lymphatic and systemic metastatic spreading [5,14,36]. Good clearance rates have been reported in refractory cases where IM was combined with isolated limb perfusion, intralesional IL-2, Bacillus Calmette–Guérin (BCG) vaccine, 5-fluorouracil or curettage [6,14,70,71,72,73,74].

RCTs are needed to better define the efficacy, dosing, schedule, and length of the treatment. The dosing schedule varies as follows: twice daily, once daily, five days/week (the most frequent) and once daily three times/week, for 8–72 weeks [6,14,70,71,72,73,74]. A complete clinical and histological regression was observed in approximately 82.3% of patients [36]. Clinical benefits may be detected after only 2 months of therapy [36].

### 4.6. Breast Cancer Skin Metastases

Breast cancer is the second most common malignancy to metastasize to the skin after melanoma [25]. BSM management is often challenging [25]. Although surgical resection and radiotherapy are the preferred treatments, BSM tend to relapse, leading to chest wall ulceration, pain and bleeding, which causes a great impact on the patient’s physical and emotional well-being [25,75].

The scientific evidence regarding the use of IM in this condition relies solely on single case reports [75]. For instance, Henriques et al. [75] successfully treated a 26-year-old woman with a triple-negative invasive ductal carcinoma with skin metastases in her left lower neck and left supraclavicular region and upper back [75]. They were refractory to systemic chemotherapy, trastuzumab, lapatinib and locoregional radiotherapy [75]. A regimen of IM 5% three times/week for four months was prescribed. The lesions partially regressed and the pain intensity was decreased [75].

### 4.7. Extramammary Paget’s Disease (EMPD)

EMPD is a rare skin malignancy that frequently arises in apocrine gland-rich anatomical regions, such as the anogenital area [5,76]. Its clinical course is often unpredictable, ranging from an indolent entity to an invasive neoplasm with locoregional and systemic dissemination [5]. The incidence is higher in patients aged 60 to 80 years [76]. It often presents as a genital plaque [76]. A better prognosis is expected if neoplastic cells are restricted to the epidermis [76]. Dermally invasive EMPD is associated with a risk of locoregional and systemic dissemination [76].

Due to its multifocal nature, aggressive surgical interventions have a high local recurrence risk [5]. For this reason, non-aggressive topical treatments such as IM are preferred, especially during the initial stages [5].

Data regarding the efficacy of IM are mostly based on case reports and series [5,76]. Sawada et al. [77] conducted a single-site nonrandomized prospective study where nine patients with in situ EMPD were enrolled. IM 5% cream was used three times per week for 6–16 weeks [77]. The product was applied in the lesions with a 1–2 cm circumferential margin [77]. The participants were assessed one month after the discontinuation of the treatment [77]. A complete clearance (clinical and histological) was achieved in five patients (56%) [77]. Nevertheless, the recurrence rate was high since three patients relapsed in the follow-up period (up to 46 months) [77]. No patient abandoned the study due to side effects [77]. Additionally, Cowan et al. [78] performed a nonrandomized prospective pilot trial study in eight patients with recurrent primary EMPD of the vulva. All of them had previously undergone partial or total vulvectomy [78]. IM 5% cream was used three times per week for 12 weeks. A complete clinical and histological response was observed in six patients (75%) by the follow-up appointment [78]. No participants progressed to invasive cancer while receiving active therapy [78]. Overall, the treatment was well-tolerated [78].

In conclusion, different treatment modalities have been used (daily to three times/week for 6–16 weeks) [5,76,77,78]. A regimen of 3–4 times/week for 6 months is the most recommended option [5,76,77,78].

### 4.8. Mycosis Fungoides

Primary cutaneous lymphomas (PCL) comprise a wide range of rare non-Hodgkin malignant monoclonal proliferations arising from skin-resident lymphocytes [79]. Cutaneous T-cell lymphomas represent the largest group of PCL (75%) [5], with mycosis fungoides (MF) being the most common form [5,79]. This disease is generally associated with an indolent clinical course [41].

IM could serve as a promising skin-directed drug for the management of cutaneous T-cell lymphomas at initial stages or even of plaques refractory to conventional treatments, such as psoralen+UVA or retinoids [5,80]. To the best of our knowledge, there are only two prospective studies that have evaluated the efficacy and safety of IM 5% cream in MF [41,81].

Deeths et al. [81] assessed the effectiveness of IM in six patients diagnosed with stable MF (stages IA-IIB). IM 5% cream was applied to a maximum of five lesions, three times per week for three months [81]. Three participants concomitantly received systemic therapy (photochemotherapy (*n* = 2) and systemic retinoids (*n* = 1)) [81]. All patients except one experienced some degree of clinical improvement [81]. The lesions were completely cleared in three participants, which was confirmed in the follow-up biopsy one month after the discontinuation of the treatment [81].

In a double-blind placebo-controlled RCT conducted by Chong et al. [41], four male patients with stage IB MF (T2N0M0) were treated with IM 5% cream once daily for 16 weeks [41]. The target area measured approximately 20 cm^2^. Simultaneously, a distant control area was chosen [41]. At week 32, the lesions treated with IM showed a mean decrease in surface area of 8.9% (vs. 39.9%) [41]. The treatment was well-tolerated [41].

Subsequently, several case reports have been published that indicated a possible benefit of IM in the management of MF [80]. Most patients had limited skin involvement, with solitary patches or plaques not ideally suited for systemic treatments [41,80,82,83]. Different regimens were employed: once daily, every other day, and five times weekly [41,80,82,83]. The treatment’s duration ranged from two weeks to six months [41,80,82,83]. The follow-up periods ranged from six to ten months [41,80,82,83]. However, future RCT with larger samples and longer follow-up periods are warranted to confirm these findings.

## 5. Adverse Reactions

IM has an overall good safety profile [5]. Most adverse reactions are mild to moderate, are easily manageable and do not require the discontinuation of the treatment, which only occurs in 2–3% of the cases [1,8]. Up to a third of patients might need pharmacological therapy to mitigate the side effects [45].

Nearly every single patient develops a local reaction consisting of a variable degree of erythema, and scaling in the treatment area [1]. In severe cases, they can be accompanied by erosions, ulceration, crusting and pain [1]. Irritation may even extend to the surrounding areas [11]. Interestingly, these inflammatory side effects occur only in previously damaged or pathological cutaneous tissue [1]. When IM is applied to healthy skin, it has been found to be no more irritating than a moisturizing lotion [1].

Since these adverse reactions are dose- and time-dependent, balancing its efficacy and tolerance is critical for assuring an adequate compliance on the patient’s part [15,20]. If significant inflammation develops, the frequency of application can be reduced, which is needed in approximately 16% of the cases [11].

It is controversial whether the severity of the side effects is associated with a better clinical and histological response [15,34]. Several risk factors for intense local reactions have been described, such as low Fitzpatrick’s phototypes (I-II), severe actinic damage and being of the female sex [1].

Apart from the classical side effects, other rarer complications have been identified [1,5,14], as follows:

- Scarring and hypopigmentation have been reported in isolated cases, especially in high-frequency regimens [5]. Nevertheless, the evidence on this topic is contradictory since patients treated with IM in AK studies showed an improvement in scarring and pigmentary scores after the treatment [1].
- Cytokine-release syndrome has seldom been noted and has been attributed to a larger synthesis and systemic release of IFN and other inflammatory mediators [1]. Its severity correlates with the size of the treated area and the degree of the local reaction induced [1].
- Contact sensitization and the exacerbation of pre-existing eczematous conditions [14].
- Hypertrophic lupus erythematosus-like reaction [21], which might be caused by the activation of plasmacytoid dendritic cells through TLR binding [21].
- Other autoimmune disorders, such as *pemphigus foliaceus,* psoriasis, autoimmune spondyloarthropathy and vitiligo [11,14].
- Angioedema [11,14].
- *Erythema multiforme* [11,14].
- Eruptive epidermoid cysts [11,14].
- Schönlein–Henoch purpura [11,14].
- Chronic neuropathic pain [11,14].

After its approval, there was a safety concern regarding the use of IM in transplanted hosts [84]. Due to an increase in IFN levels, it was hypothesized that the exposure to IM could lead to an increased risk of allograft rejection [84]. In double-blind, single-centre placebo RCT, 21 immunosuppressed renal transplant recipients were treated with IM 5% three times/week for 16 weeks for AK and viral warts [84]. None of the patients treated with IM had a deleterious effect on their renal allograft in the 1-year follow-up [84]. Nevertheless, a reduced efficacy was observed when compared to that of studies concerning immunocompetent hosts [84]. Higher-frequency regimens and combinations with other therapies should be taken into consideration in the clinical setting.

## 6. Conclusions

IM is a topically self-applied IRM that strongly activates the innate immune system and fosters a tumour-targeted T-cell response. Its mechanism of action is nonetheless pleiotropic since it displays direct antineoplastic effects through the stimulation of apoptosis, autophagy and mitochondrial disfunction. IM could represent a solid alternative to surgical resection in certain cases of skin cancer. Due to its non-aggressive nature, it preserves the cosmesis and functionality of critical areas better. Its side effects are often mild, predictable, and easily manageable. Although several case series and observational studies underline its efficacy in off-label indications, such as LM or nBCC, more RCTs are needed to confirm these findings and better define the optimal regimens.

## Figures and Tables

**Table 1:** Skin cancer treatment modalities [15,16,17,18].

Surgery	− Mohs micrographic surgery (gold-standard treatment); − Conventional excision;
Physical therapies	− Electrodessication/curettage; − Electrochemotherapy; − Radiotherapy; − Ablative CO_2_ laser;
Topical therapies	− Imiquimod; − 5-fluorouracil; − Photodynamic therapy; − Tirbanibulin;
Intralesional therapies	− IFN-α; − Methotrexate; − 5-fluorouracil; − Bleomycin; − Papillomavirus vaccine;
Systemic therapies	− Immune checkpoint inhibitors (PD-1/PD-L1); − Hedgehog pathway inhibitors (vismodegib, sonidegib); − BRAF/MEK inhibitors; − Chemotherapy; − Others.

**Table 2:** Differences between high-risk and low-risk basal cell carcinoma [3,15].

	Low Risk	High Risk
Histological subtype	− Superficial − Macronodular	− Morpheaform − Infiltrative − Micronodular
Perineural/perivascular infiltration	No	Yes
Size	<5 cm	>5 cm
Location	Remaining	− Centrofacial − Periocular − Ears
Other	Primary naïve tumour without associated high-risk factors	− Relapsing − Immunosuppression

## Data Availability

All the data are presented in this study.

## Abbreviations

5-FU	5-fluorouracil
AK	actinic keratosis
APC	antigen-presenting cells
BCC	basal cell carcinoma
BCG	Bacillus Calmette–Guérin
BD	Bowen’s disease
BSM	breast skin metastases
bFGF	basic fibroblast growth factor
CTB	cathepsin B
CTD	cathepsin D
EMPD	extramammary Paget’s disease
DC	dendritic cells
FDA	Food and Drug Administration
FLICE	Fas-associated death domain-like IL-1β-converting enzyme inhibitory protein
G-CSF	granulocyte colony stimulating factor
GM-CSF	granulocyte and monocyte stimulating factor
HPV	human papillomavirus
IM	imiquimod
IRM	immune response modifiers
LC	Langerhans cells
LM	lentigo maligna
MF	mycosis fungoides
MHC	major histocompatibility complex
MIP	macrophage inflammatory proteins
MIS	melanoma in situ
MMP-9	matrix metalloproteinase-9
NK cells	natural killer cells
NMSC	nonmelanoma skin cancer
NOS	nitric oxide synthase
nBCC	nodular basal cell carcinoma
pDC	plasmacytoid dendritic cells
PCL	primary cutaneous lymphoma
PDT	photodynamic therapy
PTCH	protein patched homolog
RCT	randomized clinical trial
sBCC	superficial basal cell carcinoma
PAMP	pathogen-associated molecular pattern
PCL	primary cutaneous lymphoma
SCC	squamous cell carcinoma
TAAs	tumour-associated antigens
TIMP	tissue inhibitor of matrix metalloproteinase
TLR	Toll-like receptor
TRAIL	TNF-related apoptosis-inducing ligand
TSP-1	thrombospondin-1
UV	ultraviolet
VEGF	vascular endothelial growth factor
